# The Negro Exempt from Hay Fever

**Published:** 1885-07-20

**Authors:** 


**The Negro Exempt from Hay Fever.**—At the first meeting of the Baltimore Clinical Society for the current year, on October 3, 1884, Dr. John Mackenzie read an interesting report of a case of "Hay Asthma in a Negro." It is a recognized fact that the negro race is practically exempt from this disease. This was the only case which Dr. Mackenzie had ever seen or heard reported. —*Medical Bulletin.*

